# Amblyopia from the Abuse of Tobacco and Alcohol

**Published:** 1881-02

**Authors:** David Webster


### AMBLYOPIA FROM THE ABUSE OF TOBACCO AND ALCOHOL.

By DAVID WEBSTER, M D.

Probably every physician has to deal with the poisonous effects upon the nervous system of alcohol and tobacco in one form or another. And, I think, the careful medical men, when called to a case of chronic disease in the adult male, and even in some cases of acute trouble, rarely omits to inquire into his habits as regards the use of tobacco and spirits, and often finds it necessary to regulate, or to forbid entirely, the use of these drugs.

That the abuse of alcohol alone, or of alcohol and tobacco combined, may produce impairment of vision, ranging all the way from the slightest blurring to total blindness, no physician acquainted with the subject will, I think, venture to deny.

Some, however, doubt that *tobacco alone* ever causes impairment of vision. And indeed it is difficult to demonstrate, beyond all controversy, that it ever does. Such cases seem to occur very infrequently in this country, and when we meet with one of them we often find it very difficult to exclude all other causes of amblyopia.

Mr. Jonathan Hutchinson, of London, seems to have no doubt of the effect of tobacco in producing amblyopia. In the Ophthlamic Hospital reports, vol. vii., he publishes twenty-nine cases that have come under his care. Of these cases he says: "For myself, I may briefly avow that I have scrupulously investigated other possible causes, and that I feel no hesitation in believing that in most of these cases tobacco is the real one." In two of Mr. Hutchinson's cases there was white atrophy of both optic disks, with total blindness. In one case the disks were very white and the patient had only perception of light. The other cases had vision ranging from perception of light to two-thirds the normal. Mr. H. states in this article that he has long held the opinion "that when tobacco causes blindness it does so in virtue of an idiosyncracy," and that "it is by no means improbable that such idiosynsrasy will be found occasionally in several members of the same family."

As regards such family idiosyncrasies, I have seen several instances of optic nerve atrophy occuring in adult males who were brothers, but, in these cases, the disease could not be attributed to the use of tobacco, nor could any very probable cause be assigned.

In vol. viii. (*ibid.*) Mr. H. publishes tables containing the "facts as to progress" in sixty-four cases of tobacco amaurosis. "Recovery or great improvement took place in seventy-five *per cent.* In four cases the disease was arrested, sight remaining stationary after a certain degree of failure. In seven cases sight became worse while under care, and five others were quite blind when first seen, and are believed to have continued so."

The fact that so careful and so competent an observer as Mr. Hutchinson has made a diagnosis of tobacco amaurosis in so large a number of cases is, of itself, one of the strongest arguments in favor of the existence of such a disease. At the same time it would appear, that it is met with much more frequently in England than in any other country. This, according to Mr. Hutchinson, seems to be accounted for by the fact that the poorer clases of Englishmen usually smoke a kind of tobacco called *shag*, one of the strongest and worst of its forms.

[A number of cases are given illustrating the improvement of vision caused by hypodermic injections of nitrate strych.]

In nearly all the cases the impairment of sight had been *gradual*, although in one case it had been ushered in *suddenly* "after prolonged exposure of the eyes to glare on the water." In this case, however, the sight continued to deteriorate gradually after the first onset of the disease. In all the cases there was simultaneous decay of vision in both eyes. In nine cases, or in nearly *fifty per cent*, the amount of impairment was exactly the same in both eyes. The greatest difference of vision in the two eyes of any one patient was where one eye had vision 20-30 and the other 20.100.

In several cases it may be doubted whether the impairment of vision was wholly due to alcohol and tobacco-poisoning. Several of the patients had been subjects of malaria; one said that his sight began to fail during an attack of "bilious diarrhœa;" one had syphilis, but there was no evidence of any syphilitic disease of his eyes, and on the other hand he was habitually saturated with spirits and tobacco, and recovered his vision when these were cut off, and he had been under treatment for a few weeks.

In all of these cases, or nearly all, the treatment by hypodermic injections of strychnia was pursued, and generally with great apparent benefit. Our routine method of using the strychnia is this: We have put up, at a reliable apothecary's, four grains of nitrate of strychnia in an ounce of distilled water. Each minim of this solution contains one one-hundred-and-twentieth of a grain of nitrate of strychnia. We usually begin by injecting into the subcutaneous cellular tissue three minims of this solution, or one-fortieth of a grain of the drug. We then increase the amount injected by one minim daily, giving one injection a day until some of the physiological effects of the drug have been produced.

The most frequent of these we have found to be a kind of stiffening of the legs with a tendency to "kick out" in an irregular manner. We have usually kept the patient sitting for fifteen or twenty minutes after the injection, in order to observe the effect. Frequently the first symptom of any effect upon the system would be this uncontrollable tendency to step higher, or to one side, or in some direction not in accordance with the wishes of the patient while going down stairs to go out. At the same time stiffness of the muscles of the thighs would be complained of, and ssmetimes slight spasmodic twitching, or rigidity, of the muscles of the jaws or other muscles would be observed. The next most frequent symptom was vertigo, and sometimes headache. I remember that one man, who lived in Williambsburg, was attended with a sudden dizziness, and fell down on the sidewalk when he had nearly reached his home. He soon recovered his selfcontrol, however, and reached home without assistance. In a very few cases "little knotty pains" in the bowels were complained of—just such pains as I have heard persons say they experienced from drinking lager beer.

I may say, however, that although we have been using nitrate ef strychnia hypodermically for over eight years, and often in three or four different patients a day, we have never yet seen any alarming symptoms from its use, and we do not think there is much danger attendant upon its use in the way we have described. Cases have been reported, however, in which symptoms of decided strychnine-poisoning followed its use in amaurosis and amblyopia. But I have not heard of any *fatal* results.

One very disagreeable complication, however, arose in several cases, and that was a tendency to inflammation at the site of the puncture.

In one case a small abscess formed and persisted for several weeks in spite of such treatment as was employed. When this complication occurs a change in the treatment usually seems to be indicated.

We have commonly given our injections in the back of the neck, sometimes in the arm, and less frequently in the temple.

The question naturally arises, How long should we continue these hypodermic injections of strychnia? Our practice has been to continue them up to physiological effects, and then to stop them if no marked benefit has ensued. If the vision continu[illegible] to improve after physiological effects have been reached, the injections may be continued for an indefinite period—that is, long as they seem to be doing any good. The qnantity injected should be diminished, however, so as just to fall short of physiological effects.

We have, in some cases, after reaching physiological effects, stopped the strychnia injections, for a few weeks and then resumed them again, going over the same ground as at first with decided benefit.

The largest dose of strychnia I have injected in any one case was, to the best of my reccollection, one-fifth of a grain. I have seen one patient, however, who, under the care of a friend of mine, had the dose increased to one-half of a grain before physiological effects were produced. Usually physiological effects show themselves after you have increased the dose to from one-fifteenth to one-tenth of a grain.

You must not suppose, gentlemen, that in these cases of amblyopia from alcohol and tobacco-poisoning we do nothing for the patient but treat him with hypodermic injections of strychnine. The first and most important of all in the treatment is *entire abstinence* from the poisons which caused the disease. We find it the best way to cut off the alcohol and tobacco *at once and completely.* The "tapering off system" will not do here. If the patient be allowed to use either tobacco or alcohol at all, he will be almost sure to do so to the point of saturation or toxic effect.

If I were to formulate my conclusions drawn from these cases and from other sources of information, they would be about as follows:

1. Amblyopia from poisoning by alcohol alone, or by alcohol and tobacco combined, is not uncommon.

2. Amblyopia from poisoning by tobacco alone *does occur*, but, in this country, somewhat rarely.

